# Cell Models for Chromosome 20q11.21 Amplification and Drug Sensitivities in Colorectal Cancer

**DOI:** 10.3390/medicina57090860

**Published:** 2021-08-24

**Authors:** Ioannis A. Voutsadakis

**Affiliations:** 1 Algoma District Cancer Program, Sault Area Hospital, 750 Great Northern Road, Sault Ste. Marie, ON P6B 0A8, Canada; ivoutsadakis@nosm.ca or ivoutsadakis@yahoo.com; 2 Section of Internal Medicine, Division of Clinical Sciences, Northern Ontario School of Medicine, Sudbury, ON P6B 0A8, Canada

**Keywords:** drug development, targeted therapies, preclinical candidates, databases, cancer therapeutics

## Abstract

*Background and objectives*: The chromosome locus 20q11.21 is a commonly amplified locus in colorectal cancer, with a prevalence of 8% to 9%. Several candidate cancer-associated genes are transcribed from the locus. The therapeutic implications of the amplification in colorectal cancer remain unclear. *Materials and Methods*: Preclinical cell line models of colorectal cancer included in the Cancer Cell Line Encyclopedia (CCLE) collection were examined for the presence of amplifications in 20q11.21 genes. Correlations of the presence of 20q11.21 amplifications with gene essentialities and drug sensitivities were surveyed on salient databases for determination of therapeutic leads. *Results*: A significant subset of colorectal cancer cell lines in the CCLE (12 of 63 cell lines, 19%) bear amplifications of genes located at 20q11.21. Cancer-associated genes of the locus include *ASXL1*, *DNMT3B*, *BCL2L1*, *TPX2*, *KIF3B* and *POFUT1*. These genes are all amplified in the 12 cell lines, but they are variably over-expressed at the mRNA level, compared to non-amplified lines. 20q11.21 amplified cell lines are sensitive to various tyrosine kinase inhibitors and are resistant to chemotherapy drugs targeting the mitotic apparatus and microtubules. CRISPR and RNAi dependencies screening revealed, besides the β-catenin and *KRAS* genes, a few recurrent gene dependencies in more than one cell line, including *YAP1* and *JUP*. *Conclusions*: Cell line models of colorectal cancer with 20q11.21 gene amplifications display dependencies on the presence of specific genes and resistance or sensitivity to specific drugs and drug categories. Observations from in vitro models may form the basis for clinical drug development in this subtype of colorectal cancer. Genetic lesions conferring synthetic lethality to certain drugs or categories of drugs could be discovered with this approach.

## 1. Introduction

Development of targeted therapies of cancer based on underlying molecular defects that drive carcinogenesis has improved cancer patient outcomes in recent years. New candidate drugs are often first tested in preclinical in vitro models consisting of cell lines cultured in highly artificial conditions [1]. Thus, their relevance for capturing the in vivo environment where the drugs under development will be acting is debatable. Moreover, tumors in situ consist not only of the tumor cells but also of a variety of supporting cells, as well as tumor attacking or tolerogenic immune cells and soluble factors (cytokines, chemokines and hormones) that both promote and antagonize tumor cell survival and proliferation [2]. Initial anti-cancer drug testing for identification of candidates for further development is performed with the aid of high-throughput screens, which, by their design, do not take into consideration the molecular characteristics of the tested cell lines. However, many of the tested drugs have a known molecular target, and their efficacy could be enhanced if the target is expressed and critical for the survival of neoplastic cells. In contrast, these drugs may be ineffective, even if pharmacologically and pharmacodynamically appropriate concentrations have been achieved, when the target is not expressed or is not critical in a given cancer [3]. Taking these factors into consideration, a molecularly informed drug development model starting early from the pre-clinical, in vitro phase, could aid in further clinical advancement of drugs with a drug–target paired model of development. A matched approach in the development process from early on could reduce the high attrition rates that hustle cancer drug discovery [4].

Amplification of specific loci is common in cancers and is often observed with tumor specificity, being more frequent in specific types of cancers. Perhaps one of the best-known amplifications with clinical therapeutic implications is observed in a subset of breast cancers at chromosome 17q. The locus includes oncogene *ERBB2*, encoding for HER2 receptor protein and leading to sensitivity to monoclonal antibodies and small-molecule tyrosine kinase inhibitors blocking the receptor [5]. In another cancer with high prevalence, colorectal adenocarcinoma, a commonly amplified region is at chromosome locus 20q11.21, which contains several potential oncogenic drivers and is amplified in about 10% of cases in the colorectal cancer cohort from TCGA [6]. In other cancers, amplifications of the 20q11.21 locus are rare and are encountered in 2.5% of head and neck carcinomas and in 1% to 1.5% of bladder carcinomas, small-cell lung carcinomas, esophagogastric and hepatobiliary cancers, which are the cancers with the higher prevalence of the amplification [7]. The amplified 20q11 region in colorectal cancers extends in many cases to neighboring loci, while in other cases, it is more restricted [7]. Genes that are located in the commonly amplified region include *ASXL1*, *DNMT3B*, *BCL2L1*, *TPX2*, *KIF3B* and *POFUT1*. The expression of resulting mRNA in 20q11.21 amplified colorectal cancers is variable. Some of the amplified genes are rarely over-expressed at the mRNA level, while others, such as ASXL1, KIF3B and POFUT1, are over-expressed in most amplified cancers. ASXL1 is over-expressed in 88.9% of 20q11.21 amplified colorectal cancers in the TCGA cohort, while POFUT1 is over-expressed in 90.5% of 20q11.21 amplified cases in the same cohort [7]. Genes that are over-expressed when amplified are putative drivers in the oncogenic process and may promote selection of the amplicon. This investigation examines drug sensitivity of colon and other cancer cell lines with amplification of genes at 20q11.21 for drugs that target driver genes of the amplicon. Dependencies of these cell lines to genomic alterations are also examined.

## 2. Methods

Cancer cell lines included in the current investigation constitute part of the Cancer Cell Line Encyclopedia (CCLE) collection [8]. The cBioportal Genomics Portal platform was used to probe CCLE for molecular abnormalities in colorectal cancer cell lines with amplification of genes located at 20q11.21 [9]. cBioCancer (http://www.cbioportal.org, accessed on 3 April 2021) is a user-friendly, open-access platform for genomic analysis of tumors and cancer cell lines [9]. Additionally, genomic data of colorectal cancer patients from The Cancer Genome Atlas (TCGA) study cohort [6] were analyzed using cBioportal. Subsets of cell lines and colorectal cancers with or without amplifications of genes at the 20q11.21 locus were identified. TCGA employs whole-exome sequencing to discover mutations, copy number alterations, and fusions in cohorts of patients with various types of cancer. Analysis of copy number alterations in TCGA is performed with the GISTIC (Genomic Identification of Significant Targets in Cancer) algorithm, in which a score of 2 or above denotes putative amplification of a gene [10]. An Aneuploidy Score (AS) is presented as a measure of chromosomal instability of cancers and is defined as the sum of the number of chromosome arms in each patient sample included in the study that display copy number alterations (gains or losses) [11]. A chromosome arm is considered copy number-altered based on the length of alterations, as calculated by the ABSOLUTE algorithm from Affymetrix 6.0 SNP arrays [12]. The definition of a somatic copy number alteration was set at more than 80% of the length of the arm. Alterations in 20% to 80% of a given arm length were considered inadequate to call, while chromosomal arms with somatic copy number alterations in less than 20% of the arm length were considered not altered. TCGA also includes mRNA expression analysis. The RSEM algorithm is used for normalization of mRNA expression [13].

The OncoKB knowledgebase is a database of cancer-related genes and classifies cancer-related genes as oncogenes or tumor suppressors [14]. OncoKB was scanned for any genes from the 20q11.21 locus included in the database and information was used to guide further analyses of putative drug dependencies.

The Genomics of Drug Sensitivity in Cancer (GDSC) dataset (www.cancerrxgene.org, accessed on 6 April 2021) was queried to obtain data on drug sensitivity of cell lines from colorectal cancer and other cancers with the 20q11.21 amplification [15]. Dependencies of these cell lines on specific genes was obtained from the Depmap portal that contains data from CRISPR arrays and RNA-interference arrays for CCLE cell lines [16,17]. These arrays screen cell lines for essential genes that are important for their survival and, as a result, their knock-down has a significant effect in their survival and proliferation in vitro [18,19,20].

Statistical comparisons of categorical data were carried out using Fisher’s exact test or the χ^2^ test. The Mann–Whitney U test was used to compare median values. All statistical comparisons were considered significant if *p* < 0.05.

## 3. Results

### 3.1. Cell Lines with 20q11.21 and Drug Sensitivity/Resistance In Vitro

Twelve cell lines among sixty-three colorectal cancer cell lines (19%) included in CCLE have amplifications of genes at the 20q11.21 locus, as assessed in cBioportal. All three genes from this locus that are listed as cancer-related at OncoKB knowledgebase (ASXL1, DNMT3B, BCL2L1) are amplified in the 12 cell lines (Table 1). Other genes of the locus with potential pathogenic importance in colorectal cancers, such as TPX2, KIF3B and POFUT1, are also amplified in the 12 cell lines. Another type of cancer with several cell lines displaying amplification of 20q11.21 genes is gastroesophageal carcinomas, where 14% to 15% of cell lines in CCLE carry amplifications of genes in the locus (Table 2). Interestingly, in contrast to colorectal cancer, gastroesophageal adenocarcinomas display 20q11.21 gene amplifications only in about 2.5% of clinical patient samples in TCGA gastroesophageal and gastric adenocarcinoma cohorts [21]. Similarly, non-small cell lung cancer (NSCLC) cell lines display a 15% to 18% prevalence of amplifications in 20q11.21 genes, while the prevalence of such amplifications in patients with either adenocarcinomas or squamous lung carcinomas is significantly lower [22,23]. The 20q11.21 locus amplification constitutes a recurrent copy number alteration confirmed in a pan-cancer cell line analysis performed in the GDSC database (feature cnaPANCAN363, www.cancerrxgene.org, accessed on 6 April 2021). Cancer cell lines with this recurrent copy number alteration, independently of primary type, display resistance to several currently used chemotherapy drugs, including the microtubule poisons vincristine, vinblastine and docetaxel, the topoisomerase II inhibitors teniposide and epirubicin and the DNA poisons temozolamide and actinomycin D (Table 3). In addition to microtubule inhibitor chemotherapeutics, several targeted mitotic inhibitors, including the Aurora A kinase inhibitor Alisertib, the kinesin protein family member 11 inhibitor Eg5 9814 and the CDC42BPA (CDC42 binding protein kinase alpha, also known as MRCK) inhibitor BDP-00009066, are associated with resistance in cell lines with 20q11.21 amplifications. Two inhibitors of DOT1L, an H3 histone methyltransferase with specificity for lysine 79 (H3K79), EPZ004777 and EPZ5676, also display resistance in these cell lines. A specific analysis of colorectal cancer cell lines with the 20q11.21 amplification shows that these cell lines are more resistant to several of those drugs (higher median IC_50_) than colorectal cancer cell lines without the amplification, although, due to the smaller size of the cohort, differences are not statistically significant except for Alisertib and EPZ004777 (Table 3).

In contrast to promoting drug resistance, the cnaPANCAN363 copy number alteration (20q11.21 amplification) does not confer sensitivity to any of the 185 drugs tested in the Genomics of Drug Sensitivity in Cancer database (GDSC). However, individual colorectal cancer cell lines with the amplification display sensitivities to tested drugs (Table 4). Drug categories that show sensitivity in more than one cell line with the cnaPANCAN363 feature include receptor tyrosine kinase inhibitors, inhibitors of intracellular kinases (PI3K, mTOR, MEK and PKC) and lipid metabolism enzyme inhibitors of sphingosine kinase and Stearoyl-CoA desaturase. No specific drugs display sensitivity in more than three amplified colorectal cancer cell lines, suggesting that the mechanism of sensitivity may not be related to the 20q11.21 amplification, that they all possess.

### 3.2. Increased mRNA Expression of Genes from 20q11.21 and Targeted Drugs

mRNA expression of genes at 20q11.21 in 12 colorectal cancer cell lines with 20q11.21 amplification was compared with the corresponding expression in 12 randomly selected colorectal cancer cell lines from CCLE without amplification in the locus. Among the six genes located at 20q11.21 with potential cancer pathogenesis interest, BCL2L1, POFUT1 and KIF3B were over-expressed at the mRNA level in 20q11.21 amplified cell lines compared with non-amplified cell lines (Table 5). Over-expression of amplified genes is partially overlapping with the over-expression of the genes in 20q11.21 amplified clinical samples of colorectal cancer patients, where POFUT1 but also ASXL1, and, in fewer cases, KIF3B and TPX2, are often over-expressed (Table 5).

Based on the mRNA over-expressions of genes at 20q11.21, the sensitivity of cell lines to BCL-xL inhibitors, Notch inhibitors (Notch pathway is activated by POFUT1 enzyme) and mitotic spindle inhibitors was evaluated at the GDSC. A notable drug in these categories displaying sensitivity in amplified cell lines compared to non-amplified cell lines is the BCL2 family inhibitor WEHI-539 (median IC_50_ in amplified lines 11.6 µmol versus 42.5 µmol in non-amplified cell lines, *p* = 0.04). In contrast, other BCL2 family inhibitors examined such as venetoclax and navitoclax showed no sensitivity in amplified cell lines or even a trend for resistance compared to non-amplified lines. Colorectal cancer cell lines with the 20q11.21 amplification displayed resistance to the microtubule polymerization stabilizer epothilone B, compared with non-amplified cell lines (median IC_50_ in amplified lines 0.028 µmol versus 0.003 µmol in non-amplified cell lines, *p* = 0.01). Z-LLNie-CHO, a γ secretase inhibitor of the Notch cascade, displayed a non-significant trend towards resistance in amplified cell lines (median IC_50_ in amplified lines 7.36 µmol versus 1.96 µmol in non-amplified cell lines, *p* = 0.23).

### 3.3. CRISPR Microarray Dependencies of 20q11.21 Amplified Colorectal Cancer Cell Lines

An evaluation of the CRISPR preferentially essential genes and RNA-interference screening of colorectal cancer cell lines with the 20q11.21 amplification disclosed a few recurrent genes in more than one line, that include *CTNNB1*, encoding for β-catenin, the WNT pathway transcription factor *TCF7L2* and oncogene *KRAS* (Table 6). In addition, BCL2L1, the gene JUP, encoding for Junction Plakoglobin (also called γ-catenin), and YAP1 (Yes-associated protein), encoding for a Hippo pathway nuclear regulator, are among additional recurrent dependency genes in 20q11.21 amplified cell lines. A similar CRISPR KO screen from project SCORE that excluded known core fitness genes of colorectal cancer, disclosed a few non-overlapping recurrent genes in colorectal cancer cell lines with 20q11.21 amplification, including DONSON (downstream of the SON gene, DNA replication fork stabilization factor), SNAP23 (synaptosome-associated protein 23) and HMGCS1 (3-hydroxy-3-methylglutaryl-CoA synthase 1).

## 4. Discussion

Amplifications of loci at the long arm of chromosome 20 are common in colorectal cancers. Genes at locus 20q11.21 are listed among the most commonly amplified genes in colorectal cancers. Colorectal cancers with the 20q11.21 amplification present at a similar stage with 20q11.21 non-amplified colorectal cancers and have a similar overall survival [7]. However, when metastatic, colorectal cancers with 20q11.21 amplification have a better survival compared with non-amplified metastatic counterparts. Moreover, 20q11.21 amplified colorectal cancer rarely harbor mutations in DNA damage response and mismatch repair-related genes compared with 20q11.21 non-amplified colorectal cancers, and have a lower tumor mutation burden [7]. The 20q11.21 locus harbors several cancer-associated genes with potential oncogenic properties. These include the epigenetic regulators ASXL1 and DNMT3B, the apoptosis regulator BCL2L1, the microtubule and mitotic spindle-associated proteins TPX2 and KIF3B and the enzyme fucosyl-transferase POFUT1. Proteins encoded by cancer-associated genes at 20q11.21 are expressed in most cases of colorectal cancers in the Human Protein Atlas [24]. Variability in the intensity of staining is observed that may underline differences in translation, in addition to gene dosage. Amplification of one or more of these genes, as a consequence of 20q11.21 locus amplification, may be the event that favors selection of the amplification and results in its comparatively high prevalence in colorectal cancer and colorectal cancer cell lines. In addition, such a driver event could be a therapeutic target for the subset of cancers carrying the amplification. Targeting a driver defect in the specific subsets of cancers that bear it would be effective in these patients and avoid treatment toxicity in the rest of the patients with no amplification of the locus. Moreover, it would help the development of targeted drugs, as a therapeutic benefit would be difficult to discern if the target population in trials is diluted by non-responders. With these considerations, the current investigation sought to take advantage of databases of in vitro cancer models in an attempt to provide a framework of sensitivities for colorectal cancers with the 20q11.21 amplification. The main findings of the current study are manifold. First, it was shown that a subset of colorectal cancer cell lines bear the 20q11.21, capturing the corresponding colorectal cancer biology of a subset of patients. Second, as a group, cancer cell lines with 20q11.21 amplifications tend to be more resistant to microtubule inhibitors, topoisomerase II inhibitors and some DNA alkylators. In addition, resistance to targeted Aurora A kinase inhibitors and kinesin inhibitors is observed in these cell lines. In contrast, the amplification does not endow 20q11.21 amplified colorectal cancer cell lines with sensitivity to any of the drugs checked in the database. However, individual cell lines with the amplification display sensitivity to assayed drugs, including kinase inhibitors and lipid metabolism inhibitors, albeit only in a few cell lines in each case. Recurrent dependencies of cell lines with the amplification include the genes for YAP1, JUP (γ-catenin), BCL2L1, DONSON, SNAP23 and HMGCS1. These dependencies may provide clues for additional therapeutic interventions. 

Amplification of 20q11.21 is observed in a significant minority of cell lines beyond colorectal cancer, such as esophagogastric and non-small lung cancers, despite the low prevalence of the amplification in patient samples from these cancers. This may suggest that the amplified segment genes confer advantage in these cancers in vitro, which is not essential in vivo. In contrast, in colorectal cancers, 20q11.21 amplifications are advantageous both in vitro and in vivo. Drug sensitivities and resistance mechanisms stemming from over-expression of genes amplified from the locus would be expected to present independently of cell line origin. With this rationale, and in order to increase statistical power from an increased number of cell lines, this study compared all 20q11.21 amplified cell lines from CCLE included in the GDSC project to those without the 20q11.21 amplification. Comparisons were, then, focused on the colorectal cell lines subsets. Results were concordant in the two comparisons, although, as expected, due to smaller numbers, they were mostly not statistically significant in the latter set of comparisons.

A different approach that could help with a better targeting of therapies to appropriate subsets of patients is by categorizing colorectal cancers to genomic subsets. Colorectal cancers have been categorized according to genomic profiles into four consensus molecular subtypes (CMS1 to 4) [25]. Cancers with 20q11.21 amplifications represent a subset of the most common canonical CSM2 cancers [7]. Characteristics of the CMS2 group include left colon laterality in 77% of cases, high level of chromosomal instability, high frequency of APC mutations leading to WNT pathway activation and lower levels of MSI lesions. KRAS mutations, BRAF mutations and SMAD4 mutations are less frequent in CSM2 colorectal cancers than in other subtypes [7,26]. Thus, CMS2 cancers and, among them, cancers with 20q11.21, would be expected to respond to EGFR-targeting therapies. Data from drug sensitivity analysis of several 20q11.21 amplified cell lines concur with this assumption of sensitivity to EGFR and downstream kinase inhibitors (Table 4). However, the molecular consensus classification does not provide any additional guidance for currently available therapies, and the need for new options based on biomarkers of efficacy remains. It is reassuring that none of the chemotherapy drugs identified as being associated with resistance in 20q11.21 cancers are used clinically in colorectal cancer. The therapeutic implications of resistance to targeted mitotic inhibitors, including Aurora A kinase and kinesin inhibitors, is of interest and suggests that TPX2 and KIF3B amplifications may be involved in dysregulation of mitosis, leading to the observed mitosis inhibitors’ resistance.

Dependencies of 20q11.21 amplified cell lines on particular genes and their products as derived from CRISPR and RNAi arrays could inform development of therapies based on synthetic lethalities. Yes-associated protein 1 (YAP1), a transcription factor of the Hippo pathway, comes up in the dependency screening of 20q amplified colorectal cancer cell lines, confirming a key role of the pathway in colorectal cancer. YAP1 co-operates with transcription factors TAZ and TEAD in transcription of genes involved in proliferation following tissue damage and promoting regeneration in the gut [27]. In colorectal cancer, aberrant signals from cancer-associated pathways, such as WNT and activated KRAS, activate Hippo to promote tumor growth and metastasis [28]. It is intriguing that the apoptosis inhibitor BCL2 is among the target genes of YAP1, a fact that could contribute to YAP1 dependency in 20q11.21 amplified cancers, given that the related BCL2L1 protein is dysregulated in these cancers [29]. Thus, interruption of Hippo signaling could impede, at least partially, aberrant cancer cell signals. CMS2 cancers are characterized by WNT pathway activation and thus a downstream activation of Hippo. Similarly, the JUP gene encoding for the β-catenin homolog, junction plakoglobin (also called γ-catenin), is also shown to be a dependence gene in a subset of 20q11.21 amplified colorectal cancer cell lines. γ-catenin has parallel roles with β-catenin in cell adhesion and WNT signaling [30]. In addition to adherens junctions, γ-catenin has a role in desmosomes [31]. These data suggest that the network of proteins associated with alternative fates of Wnt signaling is an important node in 20q11.21 amplified colorectal cancers and a candidate for therapeutic interventions.

DONSON, one of the discovered dependencies present in 2 of 7 tested amplified cell lines, is a gene of unknown function, playing a role in DNA replication and the stabilization and protection of stalled replication forks. Mutations of the gene are associated with the microcephaly-micromelia syndrome [32]. In cancer, DONSON could be helpful in preventing apoptosis during aberrant DNA replication. However, the mechanism through which 20q11.21 amplified cancers and cancers with increased chromosomal instability in general could be associated with DONSON dependence remains to be unveiled. 

SNAP23, another dependency gene present in 2 of 7 tested amplified cell lines, encodes for one of the proteins of the cellular machinery for membrane fusion and exocytosis. It is also involved in cell signaling, promoting malignant cell motion, and through this mechanism, it may favor metastasis [33].

HMGCS1 is a mevalonate precursor enzyme and catalyzes the conversion of two molecules of acetoacetyl-CoA to form 3-hydroxy-3-methyl-glutaryl-CoA (HMG-CoA), the precursor of cholesterol production [34]. HMGCS1 plays a role in breast cancer stem cells, and its downregulation decreased the stem cell fraction of both luminal and basal breast cancer cells [35]. Cholesterol biosynthesis is targeted by statins, a class of cholesterol-lowering drugs, and attempts at repurposing these drugs for cancer are in progress [36]. Interestingly, drugs targeting other lipid metabolism enzymes show activity in some 20q11.21 amplified cell lines, suggesting lipid metabolism as a possible target in these cancers. Repurposing of drugs already used for other indications for well-defined subsets of colorectal cancers would present significant advantages from financial and patient safety perspectives.

## Figures and Tables

**Table 1:** Cell lines with 20q11.21 amplifications in CCLE. Twelve cell lines have amplifications of 20q11.21 genes in cBioportal. Information for the cell line characteristics is from Cell Model Passports. LN: Lymph node, MSI: Microsatellite instability, MSS: Microsatellite stable.

Name	DepMap ID	Provenance	Ploidy	Mutations/Mb	Driver Mutations	MSI Status
C2BBe1	ACH-000009	Colon primary	3.5	8.4	APC, TP53, SMAD4, CTNNB1	MSS
COLO678	ACH-000350	LN Metastasis	2.3	7.1	APC, KRAS	MSS
HT55	ACH-000926	Colon	3.4	33.7	APC, TP53, BRAF, FAT2, DNMT3A	MSS
LS1034	ACH-000252	Cecum	3.05	3.7	APC, TP53, KRAS, TCF7L2	MSS
LS123	ACH-000501	Colon	2.7	8.03	APC, TP53, KRAS, SMAD4, CTNND1	MSS
NCI-H508	ACH-000360	Abdominal wall metastasis	4.36	8.36	TP53, PIK3CA, CREBBP, RASA2	MSS
NCI-H747	ACH-000403	Cecum, LN metastasis	2.97	6.6	APC, TP53, KRAS	MSS
RCM1	ACH-000565	Rectum	3.08	9.73	TP53, APC, KRAS, FBXW7, SMAD4, FAT2, RBM10	MSS
SNU283	ACH-000708	Rectal, omental metastasis	3.5	10.9	ATM	MSS
SW1417	ACH-000236	Duke C, grade 3, primary tumor	3	7.06	APC, BRAF, TET2, EIF4G1	MSS
SW403	ACH-000820	Colon	3	NA	NA	NA
SW837	ACH-000421	Rectal	1.8	7	TP53, APC, KRAS, FBXW7, AMER1, ACVR2A	MSS

**Table 2:** Number of cell lines from different types of cancers with amplifications of genes located at chromosome 20q11.21. In parentheses are percentages of the total number of cell lines from each type of cancer included in CCLE.

Gene	Total	NSCLC	Esophagogastric	Colorectal	Melanoma	Pancreatic	Breast	Ovarian
*ASXL1*	96 (9.3)	15 (15.6)	14 (14.6)	12 (12.5)	7 (7.3)	7 (7.3)	6 (6.3)	6 (6.3)
*BCL2L1*	109 (10.6)	20 (18.3)	17 (15.6)	12 (11)	7 (6.4)	7 (6.4)	6 (5.5)	5 (4.6)
*DNMT3B*	86 (8.3)	13 (15.1)	12 (14)	12 (14)	7 (8.1)	6 (7)	5 (5.8)	3 (3.5)
*TPX2*	108 (10.5)	19 (17.6)	17 (15.7)	12 (11.1)	7 (6.5)	7 (6.5)	7 (6.5)	5 (4.6)
*KIF3B*	97 (9.4)	15 (15.5)	14 (14.4)	12 (12.4)	8 (8.2)	7 (7.2)	6 (6.2)	6 (6.2)
*POFUT1*	98 (9.5)	15 (15.3)	14 (14.3)	12 (12.2)	8 (8.2)	7 (7.1)	6 (6.1)	6 (6.1)

**Table 3:** Drug resistance associated with amplifications of 20q11.21 (feature cnaPANCAN363) in the whole CCLE cohort of cell lines, independently of primary type. In parentheses are respective values for the colorectal cancer cell lines cohort. Bold denotes significant in both the pan-cancer and the colorectal cancer cohort. IC_50_: Inhibitory Concentration 50%.

Drug	Mechanism of Action	Group with cnaPANCAN363 (Median IC_50_ in µmol)	Group without cnaPANCAN363 (Median IC_50_ in µmol)	*p*-Value
Vinblastine	Microtubule poison	0.072 (0.27)	0.016 (0.019)	4 × 10^−7^ (0.17)
Vincristine	Microtubule poison	0.78 (0.93)	0.1 (0.24)	0.00001 (0.6)
Docetaxel	Microtubule poison	0.021 (0.05)	0.0009 (0.01)	6 × 10^−6^ (0.09)
Teniposide	Podophylotoxin, Topoisomerase II inhibitor	4.25 (9.4)	1.41 (4.4)	3 × 10^−6^ (0.15)
Epirubicin	Topoisomerase II inhibitor	0.65 (2.2)	0.31 (0.47)	9 × 10^−7^ (0.13)
Dactinomycin	DNA intercalator	0.017 (0.018)	0.007 (0.009)	0.00002 (0.6)
Temozolamide	Alkylating agent	616 (1181)	370 (626)	2.9 × 10^−7^ (0.11)
Fulvestrant	SERD	30.88 (51.73)	17.38 (26.12)	9.4 × 10^−8^ (0.16)
Nutlin-3a	Apoptosis sensitizer	275.3 (442.9)	112.9 (197.1)	5.9 × 10^−8^ (0.06)
Alisertib	AURKA inhibitor	18.5 (63.5)	5.9 (6.7)	**3 × 10^−6^ (0.01)**
EPZ004777	DOT1L inhibitor	270.2 (377.8)	162.3 (249.8)	**1.2 × 10^−7^ (0.03)**
EPZ5676	DOT1L inhibitor	461.9 (545)	243.3 (331)	5.7 × 10^−7^ (0.22)
YK-4-279	ETS inhibitor	22.1 (62.6)	7.7 (5.8)	3.6 × 10^−7^ (0.056)
Gallibiscoquinazole	?	24.5 (27.6)	12.1 (12.9)	3.6 × 10^−7^ (0.22)
Pevonedistat	NEDD8 activating enzyme inhibitor	7.74 (8.1)	1.61 (3.11)	9.7 × 10^−7^ (0.56)
BDP-00009066	MRCK inhibitor	15.97 (12.1)	9.71 (10.6)	2 × 10^−5^ (0.36)
Eg5 9814	KSP11 kinesin inhibitor	0.1 (0.57)	0.03 (0.05)	4.6 × 10^−7^ (0.11)
AGI-5198	IDH inhibitor	177.3 (184.9)	94.3 (114.4)	6 × 10^−8^ (0.09)

**Table 4:** Top-5 drugs with z-scores in cell lines with 20q11.21 amplifications. Data are from Genomics of Drug Sensitivity in Cancer datasets (GDSC1 and GDSC2, www.cancerrxgene.org, accessed on 6 April 2021).

Cell Line	Drug Name	Drug Targets	IC_50_ (µM)	Z Score	Dataset
C2BBe1	Selumetinib	MEK1, MEK2	0.19	−2.78	GDSC1
	AZD8186	PI3Kalpha, PI3Kbeta	0.90	−2.72	GDSC2
	SB505124	TGFBR1, ACVR1B, ACVR1C	0.43	−2.63	GDSC2
	TGX221	PI3Kbeta	4.99	−2.09	GDSC1
	Vismodegib	SMO	13.99	−2.03	GDSC1
COLO678	Selumetinib	MEK1, MEK2	0.91	−1.73	GDSC1
	Cetuximab	EGFR	121.42	−1.25	GDSC1
	Afatinib	ERBB2, EGFR	0.45	−1.14	GDSC1
	Selumetinib	MEK1, MEK2	0.71	−1.02	GDSC1
	Dyrk1b_0191	DYRK1B	3.32	−0.96	GDSC1
HT55	Bryostatin 1	PKC	0.01	−2.58	GDSC1
	Linsitinib	IGF1R	1.67	−2.52	GDSC2
	BMS-754807	IGF1R, IR	0.02	−2.27	GDSC2
	CAY10566	Stearoyl-CoA desaturase	1.22	−2.02	GDSC1
	Picolinic-acid	Inflammatory related	38.76	−1.88	GDSC2
LS1034	Linsitinib	IGF1R	0.24	−2.46	GDSC1
	CAY10566	Stearoyl-CoA desaturase	1.02	−2.16	GDSC1
	BMS-754807	IGF1R, IR	0.06	−2.06	GDSC1
	VX-11e	ERK2	1.10	−1.86	GDSC1
	STF-62247	Autophagy inducer	18.62	−1.52	GDSC1
LS123	Acetalax		0.37	−2.68	GDSC2
	TANK_1366	Tankyrase 1/2 (PARP5a, PARP5b)	1.77	−2.13	GDSC1
	PFI-3	SMARCA2, SMARCA4, PB1	26.60	−2.00	GDSC1
	QL-VIII-58	MTOR, ATR	0.04	−1.44	GDSC1
	Sepantronium bromide	BIRC5	0.00	−1.41	GDSC2
NCI-H508	Afatinib	ERBB2, EGFR	0.04	−2.81	GDSC1
	Afatinib	ERBB2, EGFR	0.07	−2.71	GDSC2
	Gefitinib	EGFR	0.23	−2.12	GDSC1
	Pictilisib	PI3K (class 1)	0.18	−2.00	GDSC1
	MK-2206	AKT1, AKT2	0.87	−1.97	GDSC2
NCI-H747	Amuvatinib	KIT, PDGFRA, FLT3	0.93	−2.41	GDSC1
	Enzastaurin	PKCB	0.86	−2.30	GDSC1
	Selumetinib	MEK1, MEK2	0.41	−2.28	GDSC1
	Refametinib	MEK1, MEK2	0.10	−2.18	GDSC1
	Selumetinib	MEK1, MEK2	0.12	−1.94	GDSC1
RCM1	Selumetinib	MEK1, MEK2	0.46	−2.19	GDSC1
	Refametinib	MEK1, MEK2	0.18	−1.83	GDSC1
	CCT007093	PPM1D	15.57	−1.80	GDSC1
	AS605240	PI3Kgamma	1.60	−1.69	GDSC1
	BPD-00008900		16.98	−1.61	GDSC2
SNU283	NA				
SW1417	WEHI-539	BCL-XL	0.33	−2.48	GDSC2
	Sphingosine Kinase 1 Inhibitor II	Sphingosine Kinase	10.20	−2.05	GDSC1
	CHIR-99021	GSK3A, GSK3B	3.07	−1.99	GDSC1
	Navitoclax	BCL2, BCL-XL, BCL-W	0.28	−1.61	GDSC2
	SN-38	TOP1	0.00	−1.43	GDSC1
SW403	NA				
SW837	AS605240	PI3Kgamma	1.17	−1.90	GDSC1
	Dihydrorotenone		0.23	−1.49	GDSC2
	Acetalax		7.31	−1.25	GDSC2
	VX-11e	ERK2	3.49	−1.06	GDSC1
	Trametinib	MEK1, MEK2	0.07	−0.98	GDSC2

**Table 5:** Comparison of mRNA expression of genes at 20q11.21 in cell lines with and without 20q11.21 amplifications.

	*ASXL1*	*BCL2L1*	*DNMT3B*	*TPX2*	*POFUT1*	*KIF3B*
Median cell lines with 20q11.21 amplification	19.51098	114.1379	2.201885	102.2259	32.98115	24.43462
Median cell lines without 20q11.21 amplification	16.05503	52.77	3.028	78.56	18.96	17.97
U	53	31	65	43	28	31
*p* (critical value)	>0.05 (37)	<0.05 (37)	>0.05 (37)	>0.05 (37)	<0.05 (37)	<0.05 (37)
Z (*p*)	1.06 (0.28)	2.3 (0.01)	−0.3 (0.7)	1.6 (0.09)	2.5 (0.01)	2.33 (0.01)
Over-expression in patients (%)	88.9	43.4	58.5	69.8	90.5	79.2

**Table 6:** CRISPR and RNAi dependencies of colorectal cancer cell lines with 20q11.21 amplification. CRISPR data are from CRISPR (Avana) Public 21Q1 screen and RNAi data are from a combined dataset from Board Institute, Novartis and Marcotte et al. [20], as compiled in the Cell Model Passports site (cellmodelpassports.sanger.ac.uk, accessed on 6 April 2021). NA: Not available. Recurrent genes are in bold.

Cell Line	Top-10 CRISPR Preferentially Essential Genes	RNAi Screen
C2BBe1	**JUP**, NXT1, PPIL1, KDSR, IER3IP1, SCAP, BCAS2, ERBB2, ARF4, EGFR	**CTNNB1**, **JUP**, BCAS2, URI1, STK32A, TACR3, DOT1L, SRPK1, CMAS, MED6
COLO678	**CTNNB1**, **TCF7L2**, FAM50A, PSMB6, RPL37, CCDC86, NUP43, OGDH, YPEL5, MMS22L	NXF1, **CTNNB1**, CDC40, SNRPF, VPS28, **KRAS**, DDB1, SF3A1, TSG101, RANBP2
HT55	**CTNNB1**, IRS2, **TCF7L2**, TUBB4B, ERMP1, FASN, NXT1, ACTB, IQGAP1, STXBP3	**CTNNB1**, CAPZB, ABCE1, MAGOHB, FASN, DARS1, CFL1, SRSF3, VARS1, NXT1
LS1034	NA	NA
LS123	NA	NA
NCI-H508	MYBL2, SLC22A20P, TYMS, ANKRD20A19P, SKP1, PSMA3, **CTNNB1**, **YAP1**, EFCAB8, EGFR	NA
NCI-H747	FERMT1, VPS4A, VPS4B, **KRAS**, RAB6A, NONO, SCAP, SNRPB2, **YAP1**, RHOA	**YAP1**, **KRAS**, **JUP**, UBC, **BCL2L1**, BUB1B, FERMT1, ATP6V1B2, GPX4, UBA1
RCM1	NA	NA
SNU283	NA	NA
SW1417	CDC40, PPIE, KHSRP, EFCAB8, PPP2CA, PPWD1, EIF4A3, MED11, OR56B1, CAPZB	NA
SW403	MOCS3, GRB14, LONP1, QRFPR, FKBP1A, C2orf50, ADTRP, GNB1L, SNAP91, BOC	**KRAS**, **CTNNB1**, GPX4, USP5, WDR18, **BCL2L1**, UFD1, KDM2A, TSPAN7, FASN
SW837	NF2, INTS6, SMARCA4, **KRAS**, **JUP**, ATP7A, MED16, CAB39, DBF4, TSC2	MED1, RBM19, GTF3A, CRNKL1, SMARCA4, **KRAS**, **YAP1**, TRERF1, CDC7, SLBP

## Data Availability

No data additional data related to this report are available.

## References

[1] Wilding J.L., Bodmer W.F. (2014). Cancer cell lines for drug discovery and development. Cancer Res..

[2] Goto T. (2020). Patient-Derived Tumor Xenograft Models: Toward the Establishment of Precision Cancer Medicine. J. Personal. Med..

[3] Ruiz-Moreno A.J., Torres-Barrera P., Velázquez-Paniagua M., Dömling A., Velasco-Velázquez M.A. (2018). Guide for Selection of Relevant Cell Lines During the Evaluation of new Anti-Cancer Compounds. Anticancer Agents Med. Chem..

[4] Moreno L., Pearson A.D. (2013). How can attrition rates be reduced in cancer drug discovery?. Expert Opin. Drug. Discov..

[5] Voutsadakis I.A. (2019). Chromosome 17 centromere amplification and chromosomal instability (CIN) in breast cancer: Pathogenic and therapeutic implications. Neoplasma.

[6] Cancer Genome Atlas Network (2012). Comprehensive molecular characterization of human colon and rectal cancer. Nature.

[7] Voutsadakis I.A. (2021). Chromosome 20q11.21 Amplifications in Colorectal Cancer. Cancer Genom. Proteome.

[8] Barretina J., Caponigro G., Stransky N., Venkatesan K., Margolin A.A., Kim S., Wilson C.J., Lehár J., Kryukov G.V., Sonkin D. (2012). The Cancer Cell Line Encyclopedia enables predictive modelling of anticancer drug sensitivity. Nature.

[9] Gao J., Aksoy B.A., Dogrusoz U., Dresdner G., Gross B., Sumer S.O., Sun Y., Jacobsen A., Sinha R., Larsson E. (2013). Integrative analysis of complex cancer genomics and clinical profiles using the cBioPortal. Sci. Signal..

[10] Mermel C.H., Schumacher S.E., Hill B., Meyerson M.L., Beroukhim R., Getz G. (2011). GISTIC2.0 facilitates sensitive and confident localization of the targets of focal somatic copy-number alteration in human cancers. Genome Biol..

[11] Taylor A.M., Shih J., Ha G., Gao G.F., Zhang X., Berger A.C., Schumacher S.E., Wang C., Hu H., Liu J. (2018). Genomic and Functional Approaches to Understanding Cancer Aneuploidy. Cancer Cell.

[12] Carter S.L., Cibulskis K., Helman E., McKenna A., Shen H., Zack T., Laird P.W., Onofrio R.C., Winckler W., Weir B.A. (2012). Absolute quantification of somatic DNA alterations in human cancer. Nat. Biotechnol..

[13] Li B., Dewey C.N. (2011). RSEM: Accurate transcript quantification from RNA-seq data with or without a reference genome. BMC Bioinform..

[14] Chakravarty D., Gao J., Phillips S.M., Kundra R., Zhang H., Wang J., Rudolph J.E., Yaeger R., Soumerai T., Nissan M.H. (2017). OncoKB: A Precision Oncology Knowledge Base. JCO Precis. Oncol..

[15] Iorio F., Knijnenburg T.A., Vis D.J., Bignell G.R., Menden M.P., Schubert M., Aben N., Gonçalves E., Barthorpe S., Lightfoot H. (2016). A Landscape of Pharmacogenomic Interactions in Cancer. Cell.

[16] Behan F.M., Iorio F., Picco G., Gonçalves E., Beaver C.M., Migliardi G., Santos R., Rao Y., Sassi F., Pinnelli M. (2019). Prioritization of cancer therapeutic targets using CRISPR-Cas9 screens. Nature.

[17] Boehm J.S., Garnett M.J., Adams D.J., Francies H.E., Golub T.R., Hahn W.C., Iorio F., McFarland J.M., Parts L., Vazquez F. (2021). Cancer research needs a better map. Nature.

[18] Van der Meer D., Barthorpe S., Yang W., Lightfoot H., Hall C., Gilbert J., Francies H.E., Garnett M.J. (2019). Cell Model Passports-a hub for clinical, genetic and functional datasets of preclinical cancer models. Nucleic Acids Res..

[19] Tsherniak A., Vazquez F., Montgomery P.G., Weir B.A., Kryukov G., Cowley G.S., Gill S., Harrington W.F., Pantel S., Krill-Burger J.M. (2017). Defining a Cancer Dependency Map. Cell.

[20] Marcotte R., Sayad A., Brown K.R., Sanchez-Garcia F., Reimand J., Haider M., Virtanen C., Bradner J.E., Bader G.D., Mills G.B. (2016). Functional Genomic Landscape of Human Breast Cancer Drivers, Vulnerabilities, and Resistance. Cell.

[21] Cancer Genome Atlas Research Network (2014). Comprehensive molecular characterization of gastric adenocarcinoma. Nature.

[22] Cancer Genome Atlas Research Network (2012). Comprehensive genomic characterization of squamous cell lung cancers. Nature.

[23] Cancer Genome Atlas Research Network (2014). Comprehensive molecular profiling of lung adenocarcinoma. Nature.

[24] Uhlén M., Fagerberg L., Hallström B.M., Lindskog C., Oksvold P., Mardinoglu A., Sivertsson Å., Kampf C., Sjöstedt E., Asplund A. (2015). Proteomics. Tissue-based map of the human proteome. Science.

[25] Guinney J., Dienstmann R., Wang X., de Reyniès A., Schlicker A., Soneson C., Marisa L., Roepman P., Nyamundanda G., Angelino P. (2015). The consensus molecular subtypes of colorectal cancer. Nat. Med..

[26] Lee M.S., Menter D.G., Kopetz S. (2017). Right Versus Left Colon Cancer Biology: Integrating the Consensus Molecular Subtypes. J. Natl. Compr. Cancer Netw..

[27] Guillermin O., Angelis N., Sidor C.M., Ridgway R., Baulies A., Kucharska A., Antas P., Rose M.R., Cordero J., Sansom O. (2021). Wnt and Src signals converge on YAP-TEAD to drive intestinal regeneration. EMBO J..

[28] Thompson B.J. (2020). YAP/TAZ: Drivers of Tumor Growth, Metastasis, and Resistance to Therapy. Bioessays.

[29] Jin L., Chen Y., Cheng D., He Z., Shi X., Du B., Xi X., Gao Y., Guo Y. (2021). YAP inhibits autophagy and promotes progression of colorectal cancer via upregulating Bcl-2 expression. Cell Death Dis..

[30] Nagel J.M., Lahm H., Ofner A., Göke B., Kolligs F.T. (2017). γ-Catenin acts as a tumor suppressor through context-dependent mechanisms in colorectal cancer. Int. J. Colorectal Dis..

[31] Ben-Ze’ev A., Geiger B. (1998). Differential molecular interactions of beta-catenin and plakoglobin in adhesion, signaling and cancer. Curr. Opin. Cell Biol..

[32] Karaca E., Posey J.E., Bostwick B., Liu P., Gezdirici A., Yesil G., Coban Akdemir Z., Bayram Y., Harms F.L., Meinecke P. (2019). Biallelic and De Novo Variants in DONSON Reveal a Clinical Spectrum of Cell Cycle-opathies with Microcephaly, Dwarfism and Skeletal Abnormalities. Am. J. Med. Genet. A.

[33] Williams K.C., Coppolino M.G. (2014). SNARE-dependent interaction of Src, EGFR and β1 integrin regulates invadopodia formation and tumor cell invasion. J. Cell Sci..

[34] Buhaescu I., Izzedine H. (2007). Mevalonate pathway: A review of clinical and therapeutical implications. Clin. Biochem..

[35] Walsh C.A., Akrap N., Garre E., Magnusson Y., Harrison H., Andersson D., Jonasson E., Rafnsdottir S., Choudhry H., Buffa F. (2020). The mevalonate precursor enzyme HMGCS1 is a novel marker and key mediator of cancer stem cell enrichment in luminal and basal models of breast cancer. PLoS ONE.

[36] Iannelli F., Lombardi R., Milone M.R., Pucci B., De Rienzo S., Budillon A., Bruzzese F. (2018). Targeting Mevalonate Pathway in Cancer Treatment: Repurposing of Statins. Recent Pat. Anticancer Drug Discov..

